# Histologic Chorioamnionitis and Neurodevelopment in Preterm Infants

**DOI:** 10.1001/jamanetworkopen.2025.31158

**Published:** 2025-09-09

**Authors:** Laura S. Peterson, Shalini Roy, Viral G. Jain, Stephanie L. Merhar, Karen Harpster, Nehal A. Parikh

**Affiliations:** 1 Perinatal Institute, Cincinnati Children’s Hospital Medical Center, Cincinnati, Ohio; 2 Department of Pediatrics, University of Cincinnati College of Medicine, Cincinnati, Ohio; 3 Department of Pediatrics, University of Alabama at Birmingham; 4 Neurodevelopmental Disorders Prevention Center, Cincinnati Children’s Hospital Medical Center, Cincinnati, Ohio

## Abstract

**Importance:**

Exposure to inflammation from chorioamnionitis places the fetus at higher risk of premature birth and may increase the risk of neurodevelopmental impairments, though the evidence for the latter is mixed.

**Objective:**

To evaluate whether moderate to severe histologic chorioamnionitis (HCA) is directly associated with adverse motor performance, independent of the indirect mediating effects of premature birth.

**Design, Setting, and Participants:**

This prospective, population-based cohort study recruited participants between September 16, 2016, and November 19, 2019, from referral and nonreferral neonatal intensive care units of 5 southwestern Ohio hospitals. Preterm infants of gestational age (GA) 32 weeks or younger were consecutively enrolled. The data were analyzed between January 5 and July 11, 2025.

**Exposure:**

Moderate to severe HCA vs no or mild HCA.

**Main Outcomes and Measures:**

The primary outcome was the motor score on the standardized Bayley Scales of Infant and Toddler Development, Third Edition (BSID-3), and secondary outcomes were BSID-3 cognitive and language scores. Multivariable regression analyses controlling for antenatal confounding variables were used to test the independent association of HCA with BSID-3 scores, and causal mediation analysis was used to evaluate whether premature birth or birth GA would mediate the association between HCA and neurodevelopmental outcomes.

**Results:**

A total of 304 infants (median [IQR] GA, 29.4 [27.3-31.1] weeks; 152 female [50.0%]) had complete data at developmental follow-up at corrected age 22 to 26 months. In multivariable regression analyses, infants exposed to moderate to severe HCA exhibited a decrease in BSID-3 motor scores (β estimate, −7.0; 95% CI, −11.2 to −2.8), cognitive scores (β estimate, −6.0; 95% CI, −10.2 to −1.8, and language scores (β estimate, −8.8; 95% CI, −14.5 to −3.2). Infants exposed to moderate to severe HCA were born at a median GA of 2.6 weeks (IQR, 1.2-4.0 weeks) earlier than those with no or mild HCA exposure. Causal mediation analysis showed that earlier premature birth indirectly mediated 25% of the association of HCA with BSID-3 motor scores (β = −1.7; 95% CI, −2.9 to −0.6). The remaining 75% represented a direct adverse association of HCA or inflammation with motor development (β = −5.3; 95% CI, −9.9 to −0.7). A similar mediation of premature birth was observed for HCA and cognitive scores.

**Conclusions and Relevance:**

In this prospective, regional cohort study, exposure to moderate to severe HCA in preterm infants was independently associated with adverse motor neurodevelopment at corrected age 22 to 26 months. Causal mediation analysis suggests that HCA exhibits a significantly mediated association with neurodevelopment via induction of earlier preterm birth and a direct adverse association with neurodevelopmental outcomes. These findings suggest clinical implications for parental counseling and design of anti-inflammatory therapy trials for this high-risk population.

## Key Points

**Question:**

Is histologic chorioamnionitis (HCA) associated with neurodevelopmental outcomes of premature infants (gestational age ≤32 weeks), independent of preterm birth?

**Findings:**

In this cohort study of 304 infants, moderate to severe HCA was significantly associated with adverse motor, cognitive, and language scores on standardized testing at corrected age 22 to 26 months. Mediation analyses revealed a significant direct association of HCA or inflammation with lower neurodevelopmental scores, even after accounting for the mediated indirect outcome of premature birth.

**Meaning:**

These findings suggest that moderate to severe HCA may be an independent risk factor for adverse neurodevelopment in preterm infants.

## Teaser

This cohort study evaluates whether moderate to severe histologic chorioamnionitis is directly associated with adverse motor performance in premature infants at age 2 years.

## Introduction

Preterm birth is the leading cause of perinatal morbidity and long-term neurodevelopmental impairments, including cerebral palsy and other motor, cognitive, and/or language deficits.^1^ More than half of premature births are associated with chorioamnionitis or intrauterine inflammation with or without infection.^2,3^ Clinical chorioamnionitis is diagnosed based on clinical symptoms and has a lower accuracy in diagnosing chorioamnionitis compared with histologic analysis, which involves the identification of neutrophils in the chorioamniotic membrane.^4,5,6^ Inflammation plays a crucial role in pregnancy and parturition, and as such, its dysregulation could have profound impacts on the health of both mother and fetus. The end of pregnancy signals a reversal back to the proinflammatory state, which facilitates delivery.^7,8^ Chorioamnionitis disrupts the immune regulations during pregnancy, initiating an inflammatory state that could induce preterm labor in the mother and alter immunologic homeostasis in the fetus.^8^ For this reason, there may be a higher incidence of chorioamnionitis with decreasing gestational age (GA) at birth. Because chorioamnionitis is a trigger for preterm birth, GA acts as a mediator between chorioamnionitis and neurodevelopmental impairments in that chorioamnionitis induces earlier birth, and GA is associated with adverse neurodevelopmental outcomes.^9^

The current literature is mixed about whether chorioamnionitis is directly associated with neurodevelopmental outcomes. Various studies have shown an association between chorioamnionitis and adverse neurodevelopment, including structural brain changes, delays in motor and language development, cerebral palsy, weak memory and learning functions, and cognitive deficits.^9,10,11,12,13,14,15,16,17,18^ However, other studies have found no association.^15,19,20,21,22,23^ These differences may be attributed to different ways in which chorioamnionitis is defined; whether histologic chorioamnionitis (HCA) and clinical chorioamnionitis were combined; inappropriate statistical adjustment for mediating confounders, such as GA, bronchopulmonary dysplasia, and sepsis; suboptimal statistical power; and different timing and methods for evaluating neurodevelopmental outcomes. Adjusting for these intermediate variables could result in collider stratification bias and underestimation of the total effect.^18^ Many studies incorporated or relied on clinical chorioamnionitis, which is more ambiguous than HCA.^24,25,26^ A recent study from the same cohort as this study examined the association between HCA and brain magnetic resonance imaging (MRI) abnormalities and addressed the need to account for GA as a mediator by performing a causal mediation analysis.^27^ Premature birth was found to have mediated approximately 50% of the association between HCA and MRI brain abnormalities, while the remaining 50% was a direct harmful component of the association with brain development.^18^

To address these gaps in understanding, we analyzed the association of HCA with motor, cognitive, and language outcomes at corrected age (CA) 22 to 26 months in infants born at GA 32 weeks or earlier. We hypothesize that moderate to severe HCA would be directly associated with adverse neurodevelopmental scores at CA 2 years, independent of the mediating effects of preterm birth.

## Methods

### Study Participants

In this cohort study, we examined data from the Cincinnati Infant Neurodevelopment Early Prediction Study (CINEPS), a multisite, prospective, regional cohort of preterm infants (GA ≤32 weeks). All eligible infants who received care at 5 level III or IV neonatal intensive care units (NICUs) in the Greater Cincinnati region from September 16, 2016, to November 19, 2019, were recruited. These 5 NICUs delivered care for 95% of all births GA 32 weeks or earlier within the Greater Cincinnati region during this period. We excluded infants with chromosomal or congenital anomalies affecting the central nervous system and with cyanotic heart disease. The study was approved by the Institutional Review Board of Cincinnati Children’s Hospital. Informed consent was obtained from all parents or guardians. The study followed the Strengthening the Reporting of Observational Studies in Epidemiology (STROBE) reporting guideline. The study flow diagram is shown in eFigure 1 in Supplement 1.

### Data Definitions

All placentas were sampled according to a standardized protocol across the 5 centers as described previously.^18^ Briefly, HCA (inflammation of the fetal membranes) was graded clinically by 7 trained pathologists using the criteria of Redline et al^28^; these data were documented retrospectively. Staging, which refers to the location of neutrophil infiltration, was chosen over grading to define the severity of HCA because staging is more reproducible than grading.^29^ Stage 2 and 3 HCA were considered moderate and severe, respectively (additional details are provided in the eMethods in Supplement 1).

### Assessment of Neurodevelopment

All infants were tested with the standardized Bayley Scales of Infant and Toddler Development, Third Edition (BSID-3)^30^ at CA 22 to 26 months. Research staff (K.H.) and clinicians performing the BSID-3 were trained and certified as specified by the National Institute of Child Health and Human Development Neonatal Research Network.^31^ The BSID-3 is a validated tool with a mean score of 100 (SD, 15; range, 40-160) for each of the subtests.^30^ We assigned a motor score of 46, cognitive score of 54, and/or language score of 46 for 5 children who could not be tested due to severe developmental delays. The motor composite score was our primary outcome, and cognitive and language scores were our secondary outcomes.

### Statistical Analysis

The data analysis was performed between January 5 and July 11, 2025. Descriptive statistics (maternal: age, self-reported Asian, Black, White, or other race [American Indian or Alaska Native, Native Hawaiian or Pacific Islander], and self-reported Hispanic or non-Hispanic ethnicity; infant: GA and sex) were performed using *t* tests, Mann-Whitney *U* tests, or χ^2^ tests, as appropriate. Race and ethnicity were included to describe the sample and not analyzed further. We calculated standardized mean differences between infants with and without developmental follow-up to evaluate for potential selection bias in outcome determination. To assess the independent association of HCA with BSID-3 scores, we performed multivariable linear regressions that included known a priori–selected antenatal confounding variables, including hypertensive disorders of pregnancy, antenatal corticosteroid and magnesium sulfate therapies, antenatal maternal tobacco smoking, multiple births, birth weight *z* score, sex, high-risk socioeconomic status (eg, low income, limited education), and outborn status (ie, birth at an outlying institution).

Following these multivariable models used to assess the total effect, we applied VanderWeele’s^27^ causal mediation product method to evaluate whether premature birth or birth GA would mediate the association between HCA and neurodevelopmental outcomes, treating GA as a mediator rather than as a confounder. We performed 2 additional regression analyses: (1) For the outcome model, we regressed the outcome (BSID-3 motor score) on the exposure (HCA), the mediator (GA), and all covariates, and (2) for the mediator model, we regressed the mediator (GA) on the exposure (HCA) and all covariates. We defined the natural direct effect as the HCA coefficient in the outcome model that included GA. The natural indirect effect was defined as the product of the HCA coefficient in the mediator model and the GA coefficient in the outcome model. Thus, the indirect effect is the product of the effect of HCA on GA and the effect of GA on BSID-3 motor scores. We repeated these analyses for the cognitive and language scores. Note that we first ran a causal mediation model that included an exposure (HCA)-by-mediator (GA) interaction term, which was not significant; thus, mediation models were run without an interaction term.^27^ We used the causal mediation package in Stata, version 18.0 (StataCorp LLC) to run these analyses. Significance was considered at *P* < .05.

## Results

Of the original CINEPS cohort of 395 very preterm infants, 2 infants were withdrawn, 1 died, and 38 did not have placental pathology. Of the remaining infants, 311 (87.9%) returned for developmental follow-up, and 304 (85.9%) provided complete data (eFigure 1 in Supplement 1). The infants’ median (IQR) GA was 29.4 (27.4-31.1) weeks, median (IQR) birth weight was 1200 (917-1549) g, and 152 (50.0%) were female and 152 (50.0%) were male. Mean (SD) maternal age was 29.0 (5.5) years, and 7 were of Asian (2.3%), 95 of Black (31.3%), 231 of White (76.0%), and 17 of other (5.6%) race and 18 were of Hispanic (5.9%) and 286 of non-Hispanic (94.1%) ethnicity. Baseline differences between infants with moderate to severe vs no or mild HCA in maternal and neonatal characteristics are provided in Table 1.

**Table 1.** Baseline Maternal and Infant Characteristics

Baseline characteristic	Participants, No. (%)		*P* value
	Moderate to severe HCA (n = 51)	No or mild HCA (n = 253)	
**Maternal**			
Hypertensive disorders of pregnancy	11 (21.6)	117 (46.2)	.001
Age, mean (SD), y	28.0 (6.0)	29.3 (5.3)	.17
Tobacco smoking during pregnancy	8 (15.7)	29 (11.5)	.48
Antenatal magnesium sulfate	45 (88.2)	225 (88.9)	.81
Antenatal steroids	47 (92.2)	238 (94.1)	.54
Multiple gestation	6 (11.8)	108 (42.7)	<.001
High-risk socioeconomic status	11 (21.6)	43 (17.0)	.43
Race			
Asian	1 (2.0)	6 (2.4)	.07
Black	23 (45.1)	72 (28.5)	
White	30 (58.8)	201 (79.4)	
Other^a^	1 (2.0)	16 (6.3)	
Hispanic	2 (3.9)	16 (6.3)	.74
Not Hispanic	49 (96.1)	237 (93.7)	
**Infant **			
Gestational age, median (IQR), wk	27.2 (24.9-29.0)	29.7 (27.9-31.4)	<.001
Sex			
Female	27 (52.9)	125 (49.4)	.76
Male	24 (47.1)	128 (50.6)	
Birth weight *z* score, mean (SD)	0.31 (0.73)	−0.01 (0.99)	.009
Outborn status	14 (27.5)	32 (12.6)	.02
Severe IVH or white matter injury at 36 wk on head ultrasonography	6 (11.8)	16 (6.3)	.23
Sepsis (early or late onset)	10 (19.6)	28 (11.1)	.10
Necrotizing enterocolitis	5 (9.8)	10 (4.0)	.15
Moderate to severe bronchopulmonary dysplasia	18 (35.3)	42 (16.6)	.004
Severe retinopathy of prematurity	10 (19.6)	7 (2.8)	<.001
Global brain abnormality score, median (IQR)	7 (4-12)	4 (2-7)	<.001

Abbreviations: HCA, histologic chorioamnionitis; IVH, intraventricular hemorrhage.

^a^ Other races included American Indian or Alaska Native and Native Hawaiian or Pacific Islander.

Of the 304 infants with placental histology and BSID-3 scores, 93 (30.6%) had HCA, 51 (16.8%) had moderate to severe HCA, 42 (13.8%) had mild HCA, and 40 (11.7%) had funisitis. Infants exposed to moderate to severe HCA were born at a median GA of 2.6 weeks (IQR, 1.5-3.6 weeks; *P* < .001) earlier than infants with no or mild exposure to HCA. Between infants with and without developmental follow-up, 2 confounders had a standardized mean difference greater than 0.2: GA (0.44) and female sex (−0.31) (Table 2).

**Table 2.** SMDs in Cohort Baseline Characteristics of Infants With and Without Neurodevelopmental Follow-Up at Corrected Age 22 to 26 Months

Baseline characteristic	Proportion (SD)		SMD
	No follow-up (n = 46)	Follow-up (n = 304)	
**Maternal**			
Moderate to severe HCA	0.09 (0.28)	0.17 (0.37)	−0.24
Hypertensive disorders of pregnancy	0.50 (0.50)	0.42 (0.49)	0.16
Maternal age, mean (SD), y	29.11 (5.32)	29.02 (5.47)	0.02
Smoking tobacco during pregnancy	0.11 (0.31)	0.12 (0.33)	−0.04
Antenatal magnesium sulfate	0.85 (0.36)	0.89 (0.32)	−0.12
Antenatal steroids	0.98 (0.15)	0.94 (0.24)	0.20
Multiple births	0.37 (0.49)	0.38 (0.48)	−0.01
High-risk socioeconomic status^a^	0.17 (0.38)	0.18 (0.38)	−0.01
**Infant**			
Gestational age, mean (SD), wk	30.09 (2.14)	29.07 (2.50)	0.44
Female sex	0.35 (0.48)	0.50 (0.50)	−0.31
Birth weight *z* score, mean (SD)	0.01 (0.82)	0.05 (0.96)	−0.04
Outborn status	0.13 (0.34)	0.15 (0.36)	−0.06
Severe IVH or white matter injury at 36 wk on cranial ultrasonography	0.09 (0.28)	0.07 (0.26)	0.05
Sepsis (early or late onset)	0.09 (0.28)	0.13 (0.33)	−0.12
Necrotizing enterocolitis	0	0.05 (0.22)	−0.32
Moderate to severe bronchopulmonary dysplasia	0.09 (0.28)	0.20 (0.40)	−0.32
Severe retinopathy of prematurity	0	0.06 (0.23)	−0.34
Global brain abnormality score, mean (SD)	4.26 (3.50)	6.05 (5.09)	−0.41

Abbreviations: HCA, histologic chorioamnionitis; IVH, intraventricular hemorrhage; SMD, standardized mean difference.

^a^ Defined as social risk score of greater than the 90th percentile (eMethods in Supplement 1).

### Moderate to Severe HCA and BSID-3 Scores

The median CA at follow-up was 24 months (IQR, 23-26 months). Infants exposed to moderate or severe HCA had significantly lower median BSID composite scores for motor (91 [IQR, 82-97] vs none or mild, 94 [IQR, 88-100]; *P* = .01), cognitive (90 [IQR, 70-100] vs none or mild, 95 [IQR, 85-100]; *P* = .02), and language performance (89 [IQR, 62-97] vs none or mild, 94 [IQR, 79-106]; *P* = .007) at CA 22 to 26 months in unadjusted analyses (Table 3). These associations remained after adjusting multivariable models for all relevant confounders (Table 4). Exposure to HCA was associated with β estimates of −7.0 (95% CI, −11.2 to −2.8; *P* = .001), −6.0 (95% CI, −10.2 to −1.8; *P* = .005), and −8.8 (95% CI, −14.5 to −3.2; *P* = .002) in BSID-3 motor, cognitive, and language scores, respectively, independent of a priori–selected antenatal confounders. The associations remained even after key postnatal variables were added to the model (Table 4).

**Table 3.** Association Between Moderate to Severe HCA and BSID-3 Composite Scores at Corrected Age 22 to 26 Months

BSID-3 scale	BSID-3 score, median (IQR)		*P* value
	Moderate to severe HCA	No or mild HCA	
Motor	91 (82-97)	94 (88-100)	.01
Cognitive	90 (70-100)	95 (85-100)	.02
Language	89 (62-100)	94 (79-106)	.007

Abbreviations: BSID-3, Bayley Scales of Infant and Toddler Development, Third Edition; HCA, histologic chorioamnionitis.

**Table 4.** Association Between Moderate to Severe HCA With Neurodevelopmental Performance on BSID-3 Composite Scores at Corrected Age 22 to 26 Months in Unadjusted and Adjusted Models

BSID-3 scale	Unadjusted model		Model adjusted for antenatal confounders^a^		Model adjusted for additional postnatal variables^b^	
	β estimate (95% CI)	*P* value	β estimate (95% CI)	*P* value	β estimate (95% CI)	*P* value
Motor^c^	−7.5 (−11.7 to −3.3)	.001	−7.0 (−11.2 to −2.8)	.001	−6.1 (−10.3 to −2.0)	.004
Cognitive	−6.7 (−11.2 to −2.3)	.003	−6.0 (−10.2 to −1.8)	.005	−5.6 (−9.8 to −1.4)	.01
Language	−9.4 (−15.3 to −3.5)	.002	−8.8 (−14.5 to −3.2)	.002	−9.1 (−14.8 to −3.4)	.002

Abbreviations: BSID-3, Bayley Scales of Infant and Toddler Development, Third Edition; HCA, histologic chorioamnionitis.

^a^ Adjusted for hypertensive disorders of pregnancy, antenatal corticosteroid and magnesium sulfate therapies, in utero maternal tobacco smoking exposure, infant sex, multiple births, birth weight *z* score, high-risk socioeconomic status, and outborn status.

^b^ Updated models that included all the antenatal cofounders from the original model and 5 postnatal confounders, including severe intraventricular hemorrhage and/or white matter injury on cranial ultrasonography at postmenstrual age 36 weeks or discharge, necrotizing enterocolitis (stage ≥2), culture-positive sepsis, severe bronchopulmonary dysplasia, and severe retinopathy of prematurity.

^c^ Three of the 341 infants with follow-up were unable to complete their BSID-3 motor subtest because of behavioral difficulties.

### Premature Birth as a Mediator of Poor Neurodevelopmental Outcomes

In causal mediation analysis,^27^ GA indirectly mediated an estimated 25% of the association of moderate to severe HCA with BSID-3 motor scores (β = −1.7; 95% CI, −2.9 to −0.6; *P* = .003). The remaining 75% represented a direct adverse association of HCA or inflammation with motor development (β = −5.3; 95% CI, −9.9 to −0.7; *P* = .03). Gestational age mediated 25% of the association of HCA with cognitive scores (β = −1.5; 95% CI, −2.8 to −0.2; *P* = .02) (Figure).

**Figure.**
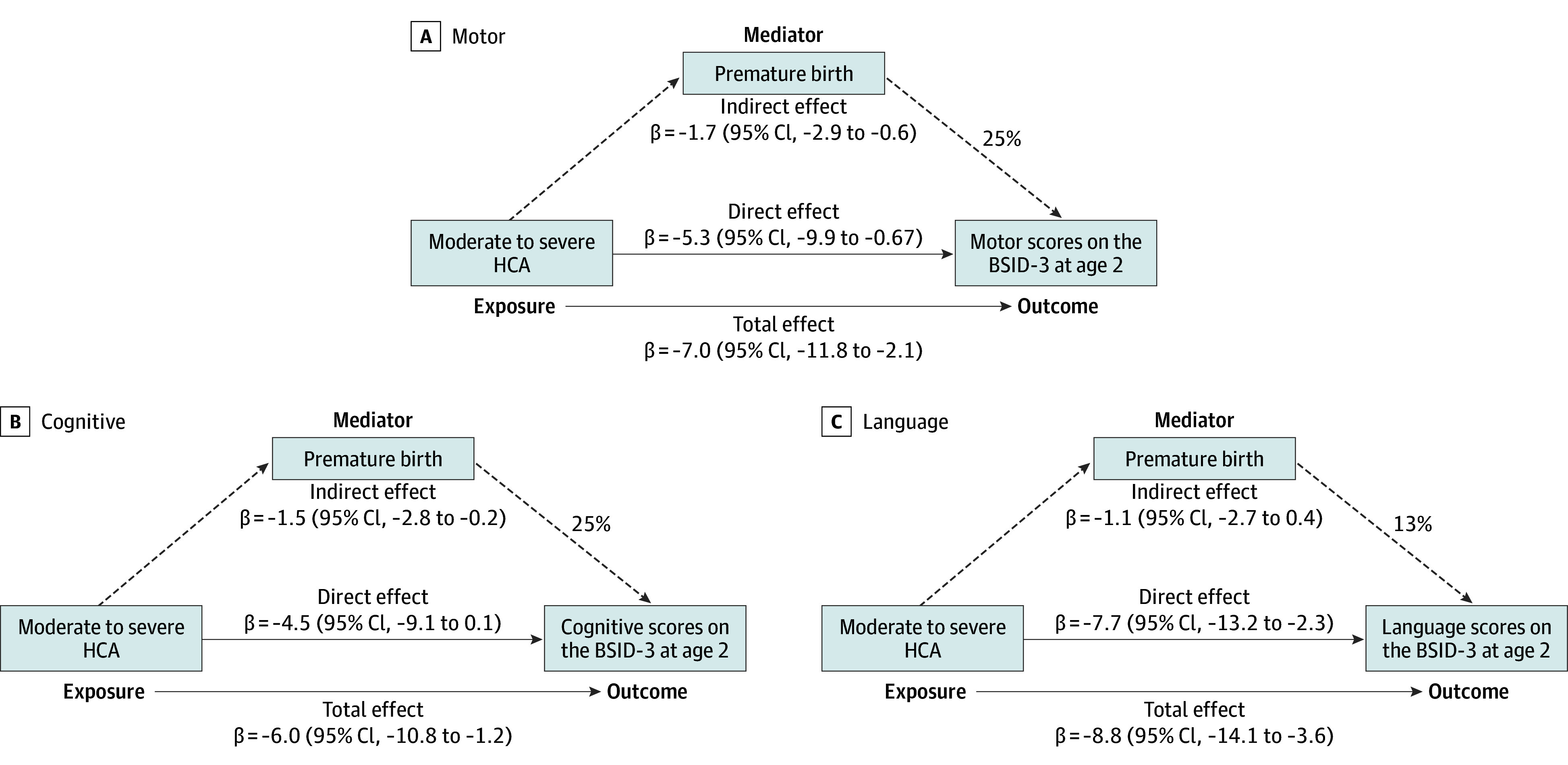
Causal Mediation Analysis of Premature Birth The directed acyclic graphs depict results of 3 mediation models to disentangle the adverse association (total effect) of moderate to severe histologic chorioamnionitis (HCA) into direct and indirect effects by accounting for the mediating effects of premature birth on the Bayley Scales of Infant and Toddler Development, Third Edition (BSID-3) scores at corrected age 22 to 26 months. Dashed lines indicate that HCA was significantly associated with BSID-3 scores at corrected age 22 to 26 months and that premature birth was a significant indirect mediator of this association.

### Global Brain Abnormalities on MRI as a Mediator of Poor Neurodevelopmental Outcomes

In causal mediation analysis, global brain abnormalities on structural MRI at term did not mediate the association of moderate to severe HCA with BSID-3 motor, cognitive, and language scores. The remaining 78% to 86% represented a direct and significant adverse association of HCA or inflammation with the BSID-3 motor (β = −5.5; 95% CI, −9.7 to −1.3; *P* = .01), cognitive (β = −4.7; 95% CI, −9.2 to −0.2; *P* = .04), and language (β = −7.6; 95% CI, −12.7 to −2.4]; *P* = .004) outcomes (eFigure 2 in Supplement 1).

In eTable 1 in Supplement 1, we present the results of the causal mediation analysis for cerebral palsy and BSID-3 scores dichotomized at less than 85 and less than 70. Only the cerebral palsy and language less-than-85 outcome model were not significant for the total effect, whereas the same model for motor and cognitive scores showed significant mediation results in which HCA showed an indirect association through preterm birth with motor and cognitive delay at CA 22 to 26 months, with risk ratios of 1.19 (95% CI, 1.01-1.40; *P* = .03) and 1.24 (95% CI, 1.06-1.46; *P* = .006), respectively. In a sensitivity analysis, the moderate to severe HCA association with BSID-3 scores (continuous) after excluding 5 children who were assigned low BSID-3 scores due to severe disability were comparable with results that included all children (eTable 2 in Supplement 1).

## Discussion

In this prospective regional cohort of preterm infants born at GA 32 weeks or earlier with perinatal exposure to intrauterine inflammation, moderate to severe HCA was independently associated with lower motor, cognitive, and language performance at CA 22 to 26 months. Moderate to severe HCA was associated with a 6- to 9-point decrease in the 3 BSID-3 scores, which equates to a 0.4- to 0.6-SD decrease on these population-normed composite scores at age 2 years. Given that moderate to severe HCA could precipitate preterm birth at earlier GAs, we performed a causal mediation analysis that confirmed our hypothesis that GA at birth partially mediates the association between HCA and motor performance, equating to approximately 25% of the total association, with the remaining 75% of the adverse association of HCA or inflammation with motor development being direct. While moderate to severe HCA also was significantly associated with total cognitive and language scores, mediation analysis only showed an indirect significant association of preterm birth with cognitive scores.

In sensitivity analyses, the addition of postnatal complications as confounders did not significantly change our original results. Like GA, postnatal complications of HCA, such as bronchopulmonary dysplasia and sepsis, may be mediators rather than confounders between HCA exposure and neurodevelopment and should not be treated as confounders in multivariable models (Table 4).

Our current results extend and validate prior findings from the same CINEPS cohort that moderate to severe HCA is associated with an increased risk of brain abnormalities at a term-equivalent age^18^ and suggest that the adverse association of HCA with neurodevelopment may be additionally mediated via development of early brain abnormalities, as indicated by our second mediation analysis. The MRI-quantified abnormalities included reductions in total brain volume; total sulcal depth; and regional volumetric and signal abnormalities in the white matter, cortical gray matter, deep gray matter, and cerebellum. This study and ours are the first to use mediation analysis to differentiate the direct associations of HCA with brain and neurodevelopmental outcomes of preterm birth. A connection between perinatal inflammation and adverse neurodevelopment has a strong pathophysiologic foundation, particularly in preterm infants. Animal studies have consistently shown that brain injury results from in utero inflammation.^2,32^ Introduction of microbes or bacterial products, such as lipopolysaccharide, into the fetal environment can both precipitate preterm labor and activate inflammatory cascades in the developing brain that inflict enduring injury.^2,33,34^ Possible mechanisms include increased permeability of cytotoxic proteins into the brain, excitotoxicity of white matter precursors, generation of reactive oxygen and nitrogen species with impairment of antioxidative pathways, and direct cytotoxic effects of cytokines. The results represent persistent microglial activation during the critical second trimester, leading not only to predominantly white matter injury but also to gray matter injury.^34,35,36,37,38^ A recent study showed a significant association among HCA, IL-8 dysregulation, and altered early white matter development in preterm infants.^39^ Indeed, large population-based studies that included mostly term or near-term infants showed that exposure to chorioamnionitis may confer increased rates of impairment, including cerebral palsy, autism, intellectual disability, and behavioral disorders.^40,41,42^

However, the current literature in preterm infants is mixed on the association between chorioamnionitis and neurodevelopment. Although several studies have agreed that chorioamnionitis has an adverse association with neurodevelopment,^9,10,11,12,13,14,15,16,17,42^ others have reported no association.^15,19,20,21,22^ Possible reasons for these discrepant results range from differences in chorioamnionitis definition, patient characteristics, and statistical approaches. Furthermore, most of the published evidence was drawn from databases of infants born decades ago, at a time when preterm infants experienced higher rates of complications and mortality. A recently published meta-analysis attempted to combine these disparate studies. Despite this heterogeneity, investigators have found that chorioamnionitis is associated with impairments in cognitive, psychomotor, and language development, particularly in cases in which the mother manifested clinical symptoms.^9^ Other data have shown a clearer association between chorioamnionitis and cerebral palsy.^41,43^ Our study may have been underpowered to examine an association with cerebral palsy.

In general, studies that have addressed the degree of severity of chorioamnionitis and included prenatal confounders have confirmed an association with neurodevelopment, though the effect size is often relatively small. A recent study by Venkatesh et al^13^ of approximately 800 extremely preterm infants (GA <28 weeks) studied the association of different stages and grades of HCA with outcomes at age 10 years. After controlling for prenatal confounders (but not GA), the authors found a complex association between severe, but not milder forms of HCA and cerebral palsy, autism, cognitive impairment, and epilepsy. Another noteworthy large study examined nearly 2000 extremely preterm infants and found that HCA accompanied by maternal clinical manifestations was associated with neurodevelopmental impairment (defined as composite indices on the BSID-2 <85 or <70) at CA 18 to 22 months.^10^ However, if the mother did not experience clinical inflammation, perhaps implying a milder case, then there was no association with impairment. Similarly, in a study by Bierstone et al^15^ of 350 infants born before GA 32 weeks, those exposed to HCA had lower BSID-3 cognitive scores in multivariable analyses (but no change in motor scores). Notably, when stratified by severity, a significant association was only observed in infants exposed to severe inflammation. However, the authors noted that incorporation of postnatal variables (sepsis, bronchopulmonary dysplasia, intraventricular hemorrhage, and white matter injury) as confounders nullified the association, although these postnatal confounders may have been alternately addressed as mediators. In contrast, in our sensitivity analyses, the association with lower BSID-3 scores persisted, even after incorporating these same postnatal variables as confounders.

Recent studies of preterm infants that did not address chorioamnionitis severity found no association with neurodevelopment. Vander Haar and Gyamfi-Bannerman^44^ retrospectively studied nearly 1500 very preterm infants born between 1997 and 2004 and found no association between chorioamnionitis and impairment on the BSID-2 test (impairment was defined as composite scores of <70 or <85), even after adjusting for prenatal confounders. However, this study defined chorioamnionitis based solely on the presence of maternal fever without placental pathology, and several relevant prenatal risk factors, such as socioeconomic factors and smoking, were not incorporated. Similarly, a large retrospective study by Miyazaki et al^22^ of nearly 6000 infants with very low birth weight born between 2003 and 2007 in Japan found no association between HCA and severe impairment at age 36 to 42 months, even after adjusting for prenatal confounders. Although this study incorporated results of placental pathology, it did not stratify based on severity of inflammation, only examined neurodevelopmental outcomes dichotomously (impaired vs not impaired), and did not include socioeconomic factors as a confounder.

Of interest, a few studies have suggested that some degree of perinatal inflammation might have a protective effect for not only neurodevelopment but also other short-term outcomes, such as survival and respiratory distress syndrome.^12,45^ Venkatesh et al^13^ found that early-stage and mild-grade chorioamnionitis was associated with a reduced odds of cerebral palsy, while advanced stage and severe grade were associated with an increased odds. These results are relatively scarce and understudied but are reminiscent of animal studies showing that mild and intermittent inflammatory or hypoxic insults could protect developing brains from subsequent events.^46,47,48^

Our study collected a rich dataset of perinatal covariates that allowed us to include and adjust for a large number of confounders known to be associated with both HCA and neurodevelopmental outcomes in preterm infants. Some have been previously included in prior studies, while others were less consistently adjusted for in the analyses. High-risk socioeconomic status and antenatal tobacco smoking are well-described risk factors, while outborn status has been variably associated with poorer neurologic outcomes.^49,50,51,52,53,54,55,56^

Our results may aid in risk stratification of preterm infants and serve as a reminder to clinicians of the long-ranging direct and indirect damage that could be caused by early-life inflammatory events, underscoring the importance of prevention of perinatal infections, when possible. Our findings also have implications for parental counseling of future neurodevelopmental impairment risk.

Further research is needed to understand the direct association of HCA with early neurostructural changes and later neurodevelopment. As we continue to follow up with our cohort over time, we hope to provide these insights. In addition, a more granular understanding of the biological underpinnings of perinatal inflammation on the developing brain may lead to better prediction, prevention, and treatments (eg, anti-inflammatory agents) for high-risk infants.

### Strengths and Limitations

Our study has several strengths. We minimized selection bias by recruiting nearly all eligible preterm infants from 5 regional level III and IV NICUs, ensuring population representation. We used prospective data collection and an objective definition of chorioamnionitis and its severity, as more than 90% of our samples had placental pathology reports. Unlike prior studies, we used causal mediation analysis to disentangle the direct association of HCA with neurodevelopment after accounting for any indirect association of early preterm birth.

 Our study also had some limitations. We did not study an inception birth cohort, so we may have underestimated the association of moderate to severe HCA with neurodevelopment or death. Additionally, BSID-3 scores at age 22 to 26 months cannot accurately estimate neurodevelopment and functioning later in life, but they do not systematically overestimate or underestimate long-term neurodevelopment.^57,58^

## Conclusions

This prospective cohort study adds to the evidence that in utero inflammation resulting from moderate to severe HCA is adversely associated with motor, cognitive, and language development in preterm infants born GA 32 weeks or earlier, independent of its indirect adverse association with neurodevelopment through preterm birth. Our findings add to the growing body of evidence that highlights in utero inflammation as an important determinant of adverse neurodevelopmental outcomes for this vulnerable population.

## Notes

Supplement 1.
eMethods.
**eFigure 1.** Flow of Participants **eFigure 2.** Causal Mediation Via Brain Abnormalities Depicted Using Directed Acyclic Graphs (DAGs) **eTable 1.** Causal Mediation Analysis Results for the Association of Histologic Chorioamnionitis (HCA) With Cerebral Palsy (CP) **eTable 2.** Sensitivity Analysis of Low Motor, Cognitive, and Language Composite Scores for 5 Children Unable to Perform Bayley Scales of Infant and Toddler, Third Edition (BSID-3) Testing
eReferences.

Supplement 2.
Nonauthor Collaborators

Supplement 3.
Data Sharing Statement
