# Supplementary material for: Histologic Chorioamnionitis and Neurodevelopment in Preterm Infants
Source: JAMA Netw Open. 2025 Sep 9;8(9):e2531158. doi: 10.1001/jamanetworkopen.2025.31158 (PMC12421339; doi:10.1001/jamanetworkopen.2025.31158)
Supplement: Supplement 1. — eMethods. eFigure 1. Flow of Participants eFigure 2. Causal Mediation Via Brain Abnormalities Depicted Using Directed Acyclic Graphs (DAGs) eTable 1. Causal Mediation Analysis Results for the Association of Histologic Chorioamnionitis (HCA) With Cerebral Palsy (CP) eTable 2. Sensitivity Analysis of Low Motor, Cognitive, and Language Composite Scores for 5 Children Unable to Perform Bayley Scales of Infant and Toddler, Third Edition (BSID-3) Testing eReferences. [file jamanetwopen-e2531158-s001.pdf]

Supplemental Online Content

Peterson LS, Roy S, Jain VG, Merhar SL, Harpster K, Parikh NA. Histologic chorioamnionitis and neurodevelopment in preterm infants. *JAMA Netw Open*. 2025;8(9):e2531158.
doi:10.1001/jamanetworkopen.2025.31158

eMethods

eFigure 1. Flow of Participants

eFigure 2. Causal Mediation Via Brain Abnormalities Depicted Using Directed Acyclic Graphs (DAGs)

eTable 1. Causal Mediation Analysis Results for the Association of Histologic Chorioamnionitis (HCA) With Cerebral Palsy (CP)

eTable 2. Sensitivity Analysis of Low Motor, Cognitive, and Language Composite Scores for 5 Children Unable to Perform Bayley Scales of Infant and Toddler, Third Edition (BSID-3) Testing

eReferences

This supplemental material has been provided by the authors to give readers additional information about their work.

© 2025 Peterson LS et al. *JAMA Network Open*.

eMethods

Placental histology

The pathologists and one of the authors (VGJ) that categorized histologic chorioamnionitis (HCA) and funisitis were blinded and unaware of any clinical risk factors or clinical outcomes. Stage 2 and 3 HCA were considered moderate and severe, respectively. Infants with no exposure to chorioamnionitis or whose placental pathology showed only stage 1 HCA were assigned to the control group. We grouped stage 1 chorioamnionitis into the control group given multiple antecedent studies suggesting mild chorioamnionitis has no or minimal impact on clinical outcomes.

Data definitions

Data were collected by trained research coordinators and included maternal demographics, characteristics of the pregnancy and delivery, and neonatal characteristics after birth and before hospital discharge using standardized definitions.[1] We calculated gestational age using the best obstetrical estimate based on the last menstrual period, early ultrasonographic examination, or other important prenatal findings, except in unusual circumstances when only an estimate by the neonatologist was availableHypertensive disorders of pregnancy was defined as maternal chronic hypertension, pregnancy induced hypertension, and/or preeclampsia, as described previously.[2] Bronchopulmonary dysplasia was classified as mild, moderate, or severe based on the categories defined by Jensen et al.[3] Severe retinopathy of prematurity was considered any retinopathy of prematurity that was stage 3 or worse, with plus disease, or that required treatment. Severe intraventricular hemorrhage was defined as grade 3 or 4 IVH based on Papile et al.[4] A dichotomous measure of high-risk social status was also utilized, which was calculated using a composite measure of six aspects of social risk status as described in Roberts et al.[5] with one difference; we replaced occupation of primary provider, which we did not collect, with household income. The composite social risk measure also includes family structure (two caregivers, separated parents with dual custody or care from another family member, single caregiver), education of primary care giver, income (≥$100,000; $40,000-$99,999; or <$40,000), employment status, language spoken at home (English, some English, no English), and maternal age. Scores could range from 0 to 12, and higher scores reflected higher socioeconomic risk. Each family was categorized as low social risk or high social risk, using a cut point of 6, which represented the 90th percentile for the social risk status in our cohort. . Our second mediator was the global brain abnormality score on structural MRI at term-equivalent age (TEA), as described by Kidokoro et al.[6] and previously used in our prior publication that linked HCA to this brain abnormalities at TEA.[7] Briefly, the global brain abnormality score is a composite score made of injury or maturational abnormalities in the white matter, cortical gray matter, deep nuclear gray matter, and/or cerebellum observed on conventional T1/T2 weighted MRI at TEA.

Statistical Analysis for Secondary Causal Mediation Analyses

In secondary analyses, we hypothesized that global brain abnormalities on structural MRI at TEA would also mediate some of the relationship between moderate-severe HCA and our three Bayley Scales of Infant and Toddler Development, Third Edition (BSID-3) subscale scores. We performed three mediation models identical to the ones for our primary analysis as stated in the main manuscript but replacing premature birth with global brain abnormalities at TEA. This analysis allowed us to disentangle the demonstrated adverse association (total effect) of moderate-severe HCA into direct and indirect effects by accounting for the mediating effects of global brain abnormalities at TEA) on the BSID-3 motor, cognitive, and language scores.

To better understand the relationship of HCA and GA with other clinically meaningful outcomes such as cerebral palsy (CP), and delays in motor, cognitive, or language development (defined as motor, cognitive, and language composite scores <85 and <70 at 22 to 26 months corrected age on the BSID-3), we performed causal mediation analysis using the "mediate" command in Stata 18.0 and using the probit command for our dichotomous outcomes. We additionally used the "estat rr" command to convert probit probabilities to relative risks (RR) to facilitate more traditional interpretation of results.

Sensitivity analyses

We performed a sensitivity analysis to facilitate comparison of our results with prior investigations that added the following postnatal covariates to the above list of confounders: severe intraventricular hemorrhage and/or white matter injury on head ultrasound at 36 weeks postmenstrual age or discharge, culture confirmed sepsis, necrotizing enterocolitis, moderate-severe bronchopulmonary dysplasia, severe retinopathy of prematurity, in addition to our a priori selected confounders. In another sensitivity analysis, we excluded five children who were unable to complete the Bayley Scales of Infant and Toddler Development, Third Edition (BSID-3) due to severe disability to determine if assigning low scores affected our results.

Results

Secondary Causal Mediation Analyses

eFigure 2 demonstrates the results of our three mediation models to disentangle the demonstrated adverse association (total effect) of moderate-severe HCA into direct and indirect effects by accounting for the mediating effects of global brain

© 2025 Peterson LS et al. *JAMA Network Open*.

abnormalities on structural MRI at TEA on the BSID-3 motor, cognitive, and language scores. Each model demonstrated that HCA associated MRI brain abnormalities was not a significant mediator but showed a trend (dashed blue lines) in the pathway between HCA and lower BSID-3 scores at 2 years' corrected age.

eTable 1 presents results of causal mediation analysis for seven outcomes, including CP, motor score<85, cognitive score<85, language score<85, motor score<70, cognitive score<70, and lanague score<70. All but the CP and language<85 models were significant for the total effect. The motor<85 and cognitive<85 models also demonstrated significant mediation results where HCA exerted an indirect effect through preterm birth on motor and cognitive delay at age 2.

Sensitivity analyses
Even after incorporating several postnatal covariates in the model, HCA remained significantly associated with lower BSID-3 scores at 22-26 months. However, as expected, HCA coefficient for the Motor model decreased as compared to our primary model without postnatal covariates(Table 4). For the second sensitivity analysis where we excluded excluding five children assigned low BSID-3 scores due to inability to complete their BSID-3 subtests due to severe disability, the results were comparable to our primary models that included these children (eTable 2).

© 2025 Peterson LS et al. *JAMA Network Open*.

eFigure 1. Flow of Participants

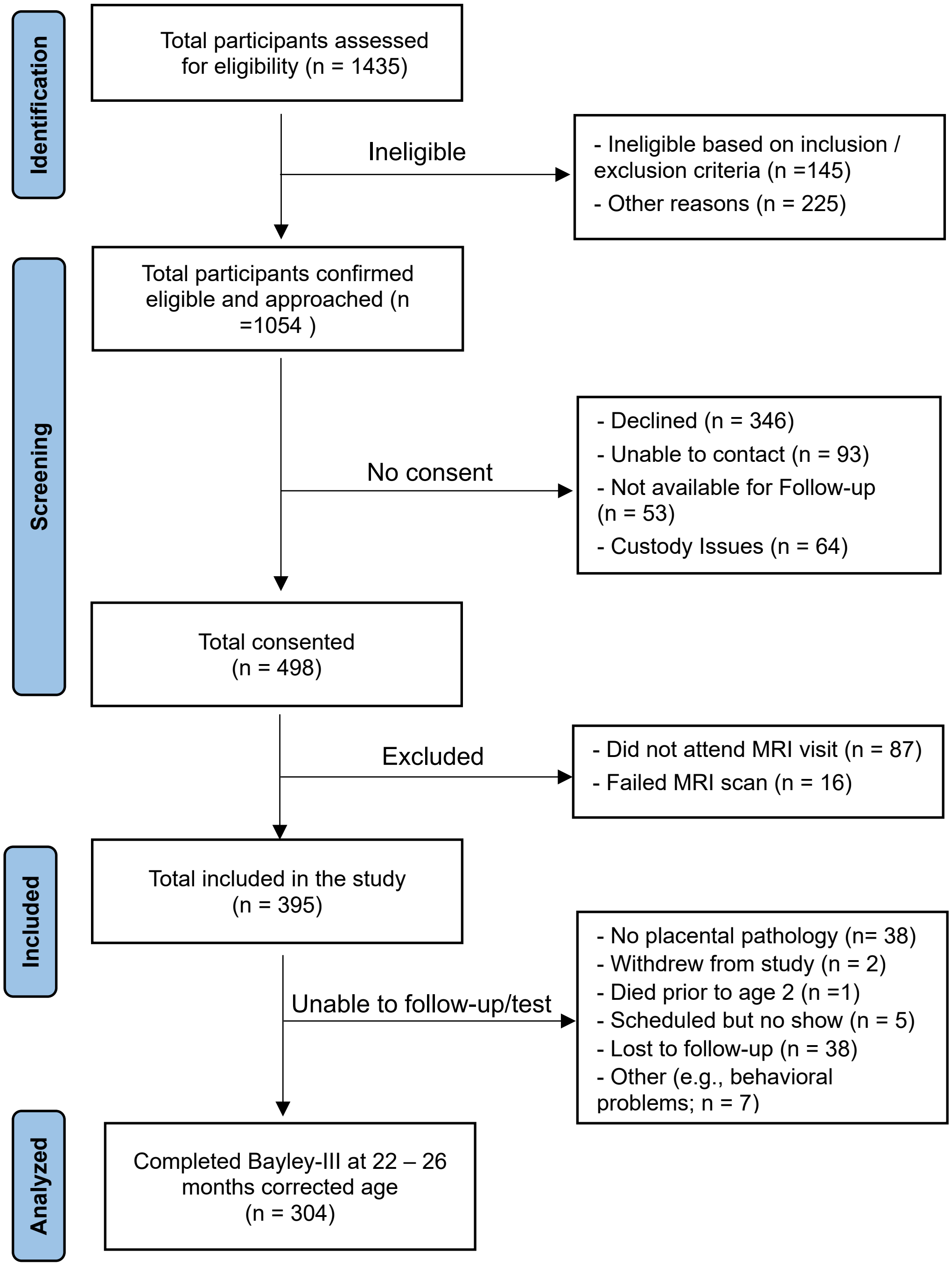

© 2025 Peterson LS et al. *JAMA Network Open*.

eFigure 2. Causal Mediation Via Brain Abnormalities Depicted Using Directed Acyclic Graphs (DAGs). Results of three mediation models to disentangle the demonstrated adverse association (total effect) of moderate-severe histologic chorioamnionitis (HCA) into direct and indirect effects by accounting for the mediating effects of global brain abnormalities on structural MRI at term-equivalent age (TEA) on the Bayley Scales of Infant and Toddler Development, Third Edition (BSID-3) (A) Motor, (B) Cognitive, and (C) Language scores. Each model demonstrated that HCA associated MRI brain abnormalities were not a significant mediator but showed a trend (dashed blue lines) in the pathway between HCA & lower BSID-3 scores at 2 years' corrected age in a regional cohort of preterm infants.

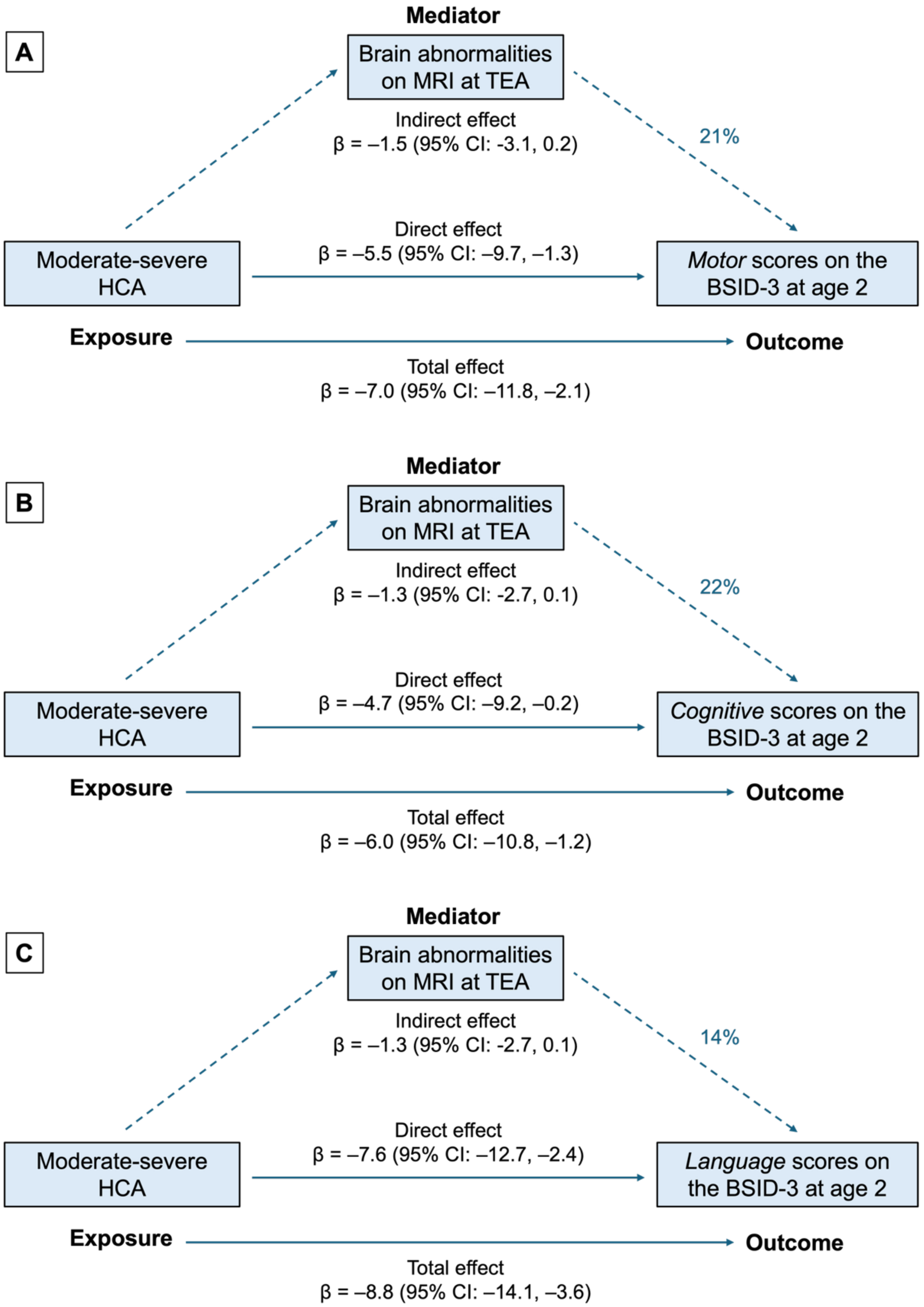

© 2025 Peterson LS et al. *JAMA Network Open*.

eTable 1. Causal Mediation Analysis Results for the Association of Histologic Chorioamnionitis (HCA) With Cerebral Palsy (CP). Causal mediation analysis results distinguishing moderate-severe HCA exposure on indirect mediated effects from premature birth/gestational age (GA) from direct association with CP and delays in motor, cognitive, and language development as determined using the Bayley Scales of Infant and Toddler, Third Edition (BSID-3) at 22-26 months corrected age in preterm

BSID-3 Outcomes*	Prevalence	Total effect of HCA		Indirect effect of GA		Direct effect of HCA		Percent Mediated
		RR (95% CI)	P value	RR (95% CI)	P value	RR (95% CI)	P value	
**CP**	11.0%	1.26 (0.58, 2.76)	0.55	NA	NA	NA	NA	NA
**Motor score <85**	18.3%	2.48 (1.53, 4.00)	*<0.001*	1.19 (1.01, 1.40)	*0.03*	2.08 (1.23, 3.51)	*0.006*	13%
**Cognitive score <85**	26.6%	1.80 (1.22, 2.67)	*0.003*	1.24 (1.06, 1.46)	*0.006*	1.44 (0.92, 2.27)	0.11	30%
**Language score <85**	33.6%	1.40 (0.97, 2.01)	0.07	NA	NA	NA	NA	NA
**Motor score <70**	5.3%	2.78 (1.07, 7.21)	*0.04*	1.29 (0.95, 1.76)	0.10	2.14 (0.78, 5.88)	0.14	NA
**Cognitive score <70**	7.2%	3.38 (1.55, 7.36)	*0.002*	1.06 (0.84, 1.34)	0.61	3.18 (1.45, 7.00)	*0.004*	NA
**Language score <70**	14.5%	2.50 (1.47, 4.26)	*0.001*	1.15 (0.97, 1.38)	0.11	2.16 (1.22, 3.83)	*0.008*	NA

infants.

*All models adjusted for hypertensive disorders of pregnancy, antenatal corticosteroid and magnesium sulfate therapies, infant sex, high risk social status, and multiple births.

eTable 2. Sensitivity Analysis of Low Motor, Cognitive, and Language Composite Scores for 5 Children Unable to Perform Bayley Scales of Infant and Toddler, Third Edition (BSID-3) Testing. Sensitivity analysis to evaluate the effect of imputing low Motor, Cognitive, and Language composite scores for five children that were unable to perform BSID-3 testing due to severe disability at 22-26 months corrected age and its association with moderate-severe histologic chorioamnionitis in preterm infants.

		Original Adjusted Models*		Models without Children Unable to Complete BSID-3 due to Severely Disability[€]	
**Exposure**	**BSID-3 Outcomes**	**β-estimate (95% CI)**	**P value**	**β-estimate (95% CI)**	**P value**
Moderate-severe HCA	Motor	-6.97 (-11.15, -2.80)	*0.001*	-5.44 (-9.57, -1.33)	*0.01*
	Cognitive	-4.48 (-8.77, -0.20)	*0.04*	-5.35 (-9.63, -1.07)	*0.02*
	Language	-8.83 (-14.46, -3.20)	*0.002*	-8.20 (-13.99, -2.40)	*0.006*

*Original model adjusted for hypertensive disorders of pregnancy, antenatal corticosteroid and magnesium sulfate therapies, in utero maternal tobacco smoking exposure, infant sex, multiple births, birth weight z-score, high risk social status, and birth at an outlying institution.
[€]Updated models excluded five children who were unable to complete the BSID-3 due to severe disability.

© 2025 Peterson LS et al. *JAMA Network Open*.

eReferences

1. Parikh NA, Sharma P, He L, et al. Perinatal Risk and Protective Factors in the Development of Diffuse White Matter Abnormality on Term-Equivalent Age Magnetic Resonance Imaging in Infants Born Very Preterm. *J Pediatr*. 2021;233:58-65.e3. doi:10.1016/j.jpeds.2020.11.058

2. Jain S, Barnes-Davis ME, Fu TT, et al. Hypertensive Disorders of Pregnancy and Risk of Early Brain Abnormalities on Magnetic Resonance Imaging at Term among Infants Born at ≤32 Weeks' Gestational Age. *J Pediatr.* 2024;273:114133. doi: 10.1016/j.jpeds.2024.114133

3. Jensen EA, Dysart K, Gantz MG, et al. The Diagnosis of Bronchopulmonary Dysplasia in Very Preterm Infants. An Evidence-based Approach. *Am J Respir Crit Care Med*. 2019;200(6):751-759. doi:10.1164/rccm.201812-2348OC

4. Bowerman RA, Donn SM, Silver TM, Jaffe MH. Natural history of neonatal periventricular/intraventricular hemorrhage and its complications: sonographic observations. *AJR Am J Roentgenol*. 1984;143(5):1041-1052. doi:10.2214/ajr.143.5.1041

5. Roberts G, Howard K, Spittle AJ, Brown NC, Anderson PJ, Doyle LW. Rates of early intervention services in very preterm children with developmental disabilities at age 2 years. J Paediatr Child Health. 2008;44(5):276-280. doi:10.1111/j.1440-1754.2007.01251.x

6. Kidokoro H, Neil JJ, Inder New MR Imaging Assessment Tool to Define Brain Abnormalities in Very Preterm Infants at Term TE.AJNR Am J Neuroradiol. 2013 Nov-Dec;34(11):2208-14.

7. Jain VG, Kline JE, He L, Kline-Fath BM, Altaye M, Muglia LJ, DeFranco EA, Ambalavanan N, Parikh NA; Cincinnati Infant Neurodevelopment Early Prediction Study Investigators. Acute histologic chorioamnionitis independently and directly increases the risk for brain abnormalities seen on magnetic resonance imaging in very preterm infants. Am J Obstet Gynecol. 2022 Oct;227(4):623.e1-623.e13.

© 2025 Peterson LS et al. *JAMA Network Open*.
