# Supplementary material for: Histologic Chorioamnionitis and Neurodevelopment in Preterm Infants
Source: JAMA Netw Open. 2025 Sep 9;8(9):e2531158. doi: 10.1001/jamanetworkopen.2025.31158 (PMC12421339; doi:10.1001/jamanetworkopen.2025.31158)
Supplement: Supplement 2. — Nonauthor Collaborators [file jamanetwopen-e2531158-s002.pdf]

Supplemental Online Content: Nonauthor Collaborators
*First name, last name, and suffix (if applicable) are required and will appear in PubMed.

***Group Name(s): Cincinnati Infant Neurodevelopment Early Prediction Study (CINEPS) Investigators**							
***First Name and Middle Initial(s)**	***Last Name**	***Suffix (eg, Jr, III)**	Academic Degrees	Institution	Location (city, state/province, country)	Role or Contribution, eg, chair, principal investigator	Group (if more than 1 Group listed in the byline) and/or Subgroup (eg, Steering Committee)
Beth	Kline-Fath		MD	Cincinnati Children's Hospital Medical Center	Cincinnati, OH, USA	Collaborator	
Jean	Tkach		PhD	Cincinnati Children's Hospital Medical Center	Cincinnati, OH, USA	Collaborator	
Hui	Wang		PhD	Cincinnati Children's Hospital Medical Center	Cincinnati, OH, USA	Collaborator	
Mekibib	Altaye		PhD	Cincinnati Children's Hospital Medical Center	Cincinnati, OH, USA	Collaborator	
