# Supplementary material for: Histologic Chorioamnionitis and Neurodevelopment in Preterm Infants
Source: JAMA Netw Open. 2025 Sep 9;8(9):e2531158. doi: 10.1001/jamanetworkopen.2025.31158 (PMC12421339; doi:10.1001/jamanetworkopen.2025.31158)
Supplement: Supplement 3. — Data Sharing Statement [file jamanetwopen-e2531158-s003.pdf]

Data Sharing Statement

Data

Data available: Yes
Data types: Deidentified participant data
How to access data: Nehal.Parikh@cchmc.org
When available: With publication

Supporting Documents

Document types: Statistical/analytic code
How to access documents: Nehal.Parikh@cchmc.org
When available: With publication

Additional Information

Who can access the data: Researchers whose proposed use of the data has been approved. Stata analysis code can be accessed from GitHub: (https://github.com/naparikh7/HistologicChorio_Neurodevelopment_JAMANetworKOpen).
Types of analyses: For any purpose
Mechanisms of data availability: After approval of a proposal
Any additional restrictions: None
